# Physical Activity and Sleep Quality in Spanish Primary School Children: Mediation of Sex and Maturational Stage

**DOI:** 10.3390/children10040622

**Published:** 2023-03-26

**Authors:** Alvaro Pano-Rodriguez, Rosa Arnau-Salvador, Carmen Mayolas-Pi, Vicenç Hernandez-Gonzalez, Alejandro Legaz-Arrese, Joaquim Reverter-Masia

**Affiliations:** 1 Department of Education Science, Faculty of Education, Psychology and Social Work, University of Lleida, 25003 Lleida, Spain; 2 Consolidated Research Group Human Movement Generalitat de Catalunya, University of Lleida, 25003 Lleida, Spain; 3 Section of Physical Education and Sports, Faculty of Health and Sport Sciences, University of Zaragoza, 50009 Zaragoza, Spain

**Keywords:** sleep quality, children, primary school, physical activity, biological maturation

## Abstract

Background: sleep is a physiological process that is critical for physical and mental health in children. Childhood encompasses diverse developmental stages that may affect the impact of physical activity on sleep quality, which may also be influenced by sex. The purpose of this study was to examine the mediation effect of sex and, as well as maturational stage on the association between physical activity and sleep quality, among primary school children. Methods: this was a cross-sectional study of 954 Spanish primary school students (437 early childhood and 517 middle childhood) with a mean age of 10.5 ± 1.2 years. Participants reported their sleep quality using the Pittsburgh Sleep Quality Index and their physical activity levels using the Physical Activity Questionnaire. Results: our study found that physical activity is associated with improved sleep quality in children, particularly during middle childhood. Higher physical activity was linked to better sleep quality and reduced sleep latency (*p =* 0.044). Sleep quality was generally better in males than in females (*p =* 0.002) and was also better in early than middle childhood (*p =* 0.000). Conclusions: especially in middle childhood, physical activity promotes children’s sleep quality. Thus, educational institutions should promote or improve the implementation of physical activity in the school context in order to benefit children’s sleep quality and, hence, improve their quality of life and wellbeing.

## 1. Introduction 

Sleep is a physiological process whose correct progress is essential for physical and mental human health. This statement acquires even more significance in children due to the special role of sleep in their healthy development and wellbeing [1]. It has a wide range of functions, which include promoting growth and development, facilitating learning and memory retention, improving the efficiency of synaptic connections, regulating behavior and emotion, strengthening the immune system, and facilitating the removal of harmful neurotoxins [2]. Research shows that there has been a deterioration in the quality of childhood sleep over the last decade, a fact that is generating certain concerns in public health institutions [3]. According to the literature, this sleep deterioration is multifactorial and is based on different aspects such as exposure to synthetic lighting, prolonged use of electronic screens during the night, consumption of caffeine, and the absence of established sleep schedules and rules within the household [4]. The consequences of this sleep loss vary from lack of focus, decreased ability to make decisions and perform tasks, and lower academic achievement to higher likelihood of developing obesity and cardio-metabolic disorders [5].

The term “sleep quality” is frequently used in sleep medicine, but it is difficult to objectively define and measure it since it represents a complex phenomenon. It may include various quantitative aspects of sleep, such as total sleep time, sleep onset latency, sleep maintenance, wake after sleep onset, sleep efficiency, and sometimes sleep disruptions such as spontaneous arousal or apnea [6]. The factors that determine sleep quality may differ among individuals. Surveys to analyze large-scale populations are commonly based on inquiries pertaining to overall habitual sleep patterns and the various types of disruptions or abnormalities in sleep. In the case of childhood, it is important to comprehend the consequences of inadequate sleep quality in order to create effective public policies and develop strategies to reduce the harmful effects of sleep deficiency. The research on this topic plays a significant part in providing information for public policies, instructions, and schools. Then, advice and suggestions should be given through interventions to educate parents and children on how to maintain healthy sleep habits. Out of the many strategies recommended to improve children’s sleep quality, physical activity is often highlighted, since it can help establish healthy sleep patterns and habits. This can include going to bed earlier, sleeping for longer durations, and enhancing sleep efficiency, which is the proportion of total time spent sleeping relative to the time spent in bed [7]. Physical activity has been linked to longer and better sleep quality for children and adolescents due to the discipline and social interaction that comes with participating in sports, as well as the significant energy expenditure and physical conditioning it provides [8]. This positive impact can improve the quality of life for children and adolescents by enhancing their sleep patterns. Research has shown that adolescents who engage in more physical activity, both as reported by themselves (subjective) and as measured objectively, are more likely to experience better sleep quality both subjectively (perception of sleep quality) and objectively (measured by scientific means) [9].

An interesting question proposed in the bibliography over the last years is the possible sex difference in sleep decline. Apparently, due to biological and sociocultural factors that influence sleep patterns, there is more impaired sleep in females. This is a phenomenon generally observed from adolescence, when hormonal events underlying puberty may be involved [10]. Nevertheless, there are conflicting results when it comes to understanding if sleep health differs between boys and girls in childhood. It is uncertain whether one gender is more or less likely to experience problems with children’s sleep [11] and that causes the necessity of further investigation regarding this issue. It still remains unknown whether there are differences or variations in the way that sex affects the interaction between physical activity and sleep quality.

Childhood includes different developmental phases. Early childhood is considered chronologically from three to nine years old, and middle childhood is from ten to eleven years old [12]. Each degree of maturational growth implies specific hormonal and skeletal characteristics [13], which could affect the relationship between physical activity and sleep quality. Nevertheless, it is currently uncertain whether the positive influence of physical activity on sleep quality is affected by the maturational growth stage. Additionally, the differences in biological growth maturation rhymes by sex could widely affect this equation. Thus, this study aims to determine the mediation of sex and degree of maturation stage on the association between physical activity and sleep quality in primary school children.

## 2. Materials and Methods

### 2.1. Design

This is a cross-sectional study based on self-report data conducted in accordance with the Declaration of Helsinki and approved by the Clinical Research Ethics Committee of the Sports Administration of Catalonia 07/02/2018/CEICGC. The data in the present study belong to the initial evaluation of a crossover study project (Searching for the “normal” reference values of biomarkers of cardiac damage after sessions of physical activity) carried out between February and May 2019.

This project was conducted by an independent group of health sciences evaluators. In order to recruit participants, primary education centers were invited to participate in this study. All school principals received an information sheet on the nature and purpose of the study. The procedure began with a concise and easily comprehensible introduction to the study, after which the participants were given a thorough explanation of the various components of the questionnaires. There was no specific time limit imposed for completing the forms, and on average, participants took approximately 40 min to complete them. Subjects and parents or legal guardians were instructed about the anonymous and voluntary nature of participation, and all of them signed informed consent.

### 2.2. Participants

All participants were recruited following the general inclusion criteria (free of any chronic disease) and exclusion criteria (being older than 12 years old). A total of 954 children (429 boys and 525 girls, 10.5 ± 1.2 years) took part in this study. The sample was formed by 437 early childhood (students in the second cycle, 9.4 ± 0.6 years) and 517 children representing middle childhood (students in the third cycle, 11.4 ± 0.6 years), belonging to 13 primary schools around the Spanish province of Lleida.

### 2.3. Outcomes

#### 2.3.1. Sleep Quality

The sleep quality was measured with the Spanish version of the Pittsburgh Sleep Quality Index (PSQI) [14]. The questionnaire serves as a dependable method for identifying sleep-related issues in both clinical and non-clinical populations, possessing high levels of reliability and validity. While its structural validity may not be exceptionally robust in all samples, the questionnaire still appears to be effective in fulfilling its intended purpose [15]. This tool was validated to assess adolescents’ sleep quality in young people [16]. It includes 19 questions on seven components of sleep quality: subjective sleep quality, sleep duration, sleep latency, habitual sleep efficiency, sleep disturbance, use of sleep medication, and daytime dysfunction. The PSQI questionnaire rates seven aspects of sleep quality using a scale that goes up in increments of three points, with zero points signifying optimal sleep quality and three points indicating suboptimal quality. The sum of all the scores is the overall PSQI score, which can range from 0 (excellent sleep quality) to 21 (very poor sleep quality). The PSQI is a highly effective tool for detecting inadequate sleep quality, with a total score of more than 5 indicating potential problems [15], similar in effectiveness to assessments conducted in clinical and laboratory settings (such as polysomnography).

#### 2.3.2. Level of Physical Activity

The level of physical activity was measured with the Spanish version of the Physical Activity Questionnaire for older children PAQ-C validated by Manchola-González (2017) [17]. The questionnaire provides a summary activity score. The PAQ-C assesses general moderate to vigorous physical activity levels carried out in the last seven days of a typical week, through 10 questions about the type and frequency of activities carried out. The questionnaire has 10 questions, and the first nine are used to calculate the final score, and the remaining question is used to identify whether the child was ill or there was some circumstance that prevented him or her from routinely performing physical activity that week. A score from 1 to 5 is obtained from the responses, with a higher score indicating greater activity. Based on their final score, the children were classified into three terciles to then perform the analysis [18].

### 2.4. Statistical Analysis

For statistical analysis, IBM Statistical Package for the Social Sciences (IBM SPSS Statistics for Windows, version 20.0; IBM Corp, Armonk, NY, USA) software was used with a statistical significance level set at α = 0.050. Descriptive statistics of the main variables were performed using mean, standard deviation, and 95% confidence interval according to sex. A one-way ANOVA was used to test the data on the relationship between the sleep quality variables according to the independent factors of sex, maturational stage, or levels of physical activity. Three-way ANOVA was used to determine if there was an interaction effect between the three independent variables on sleep quality.

## 3. Results

### 3.1. Physical Activity Levels and Sleep Quality

The descriptive values of the whole sample and the comparison of the sleep variables according to sex and maturational stage are presented in Table 1.

The results show significant differences according to sex in physical activity levels, being higher in males than females (*p* = 0.004).

Analogously, results show significant sex differences in several of the components of sleep quality, including the global score. Sleep quality is generally better in males than in females (*p* = 0.002), and this accounts for quality (*p* = 0.004), latency (*p* = 0.001), and disturbance (*p* = 0.000).

### 3.2. Interaction between Physical Activity, Sex, and Maturational Stage and Their Mediation on Sleep Quality

Results for PSQI score are shown in Table 2 by sex, by level of physical activity, and by cycle. Additionally, results are shown by sex versus cycle, sex versus physical activity, and sex versus cycle versus physical activity. Three-way ANOVA analysis showed some interaction effects of physical activity level, maturational stage, and sex in the sleep disturbances (Table 2). The maturational stage and sex were shown to influence sleep quality on their own. In males, higher levels of physical activity improve sleep disorders in mid-childhood, but not in early childhood.

It is observed an interaction between the maturational stage and physical activity in global scores and latency. In general, higher physical activity levels improve sleep quality (0.044) and latency (0.017), with the difference being somewhat greater in middle childhood.

## 4. Discussion

The current research examined how physical activity and sleep are connected among children. It also analyzed the mediation of sex and growth maturation on this connection. The main findings were that physical activity influences children’s sleep in such a way that, mainly in middle childhood, the greater the physical activity, the better the quality of sleep and less latency. Physical activity and sports had been related to bringing benefits to the quality of sleep and life of children and adolescents [13]. Nevertheless, it is in adolescents where the promotion of sleep by physical activity has been wide more studied and evidenced, since this line of research has been less developed in children. Our findings are in the same line as those of Afonso A. et al. (2022) [19], who recently found that children who accumulated at least 60 min of physical activity and sports per day went to bed earlier and had a longer sleep duration. The same study observed that children who participated in or competed in sports presented better sleep efficiency. The cause behind the clearer association between exercise and sleep quality in adolescents could be their greater growth maturation, which could let them carry out bigger amounts of exercise and consequently achieve greater levels of fatigue than children [7,8,9]. This maturation factor could be the reason why we found a stronger influence of physical activity on sleep in middle childhood than in early childhood.

The characteristics of discipline and social interaction promotion, in addition to high energy expenditure and physical conditioning, could be factors triggering this positive association between exercise and sleep in children, and this synergy seems to be highly beneficial for children’s health by improving their body composition. This was constated by Stone M.R et al. (2013), who observed that children who slept for less than nine hours were less active overall and had a higher likelihood of being overweight or obese compared to those who slept for ten or more hours (*p* < 0.05) [20]. Other factors decisive in the healthy life of children, such as screen time and sedentary behavior, have been observed to play a transcendental role in this association between physical activity and sleep quality [21].

The observed improvement of sleep latency under the influence of regular physical activity in middle childhood is in line with those results found all around the bibliography according to the meta-analytic review of Kredlow M.A. et al. (2015) [22]. The authors found that engaging in regular exercise had a somewhat positive impact on the time it took to fall asleep, suggesting that individuals who maintained a consistent exercise routine experienced significantly better sleep onset latency than those who did not exercise regularly. According to their results, this phenomenon happened specifically to younger individuals. The effect they found of regular exercise on sleep latency (d = 0.75) is similar to the outcomes of the meta-analysis of Smith et al. (2002) [23], which evaluated pharmacological and behavioral treatments for insomnia: The mean effect sizes, based on data from subjective sleep diaries, ranged from moderate to very large for sleep onset latency (d = 0.45 for pharmacotherapy and d = 1.05 for behavioral therapy). The cause explaining this positive impact could be that the energy expenditure that physical activity implies could bring as a consequence higher levels of fatigue at the time to go to bed, which would accelerate the moment of sleep commencement.

Females showed worse sleep quality than males (*p* = 0.001). Contrary to our results, some studies using self-reported methods observed that male children had worse sleep quality attending factors, such as shorter sleep duration [24,25,26], and those factors are attributed to differences in social and nutritional factors. Nevertheless, other studies observed results along the same line as ours, finding that female children were more likely to have poorer sleep quality [27], including shorter sleep duration [28], more difficulty waking up [29], higher daytime sleepiness [30], and more irregular sleep between a week and weekend nights [29]. Causes of these sleep sex differences could be the higher internalization of problems that females experience during their youth [31]. The way females assumed the social role assigned to them and perhaps their greatest concern for being accepted by their peers [32] could affect their sleep more than it does in their counterparts. Anyway, the different results observed on how sleep health in childhood is affected by gender contribute to the need for future studies to explore and understand the underlying factors that contribute to these gender differences. This could involve examining potential biological, social, and environmental influences that may affect sleep health in boys and girls differently, as well as investigating any potential cultural or societal factors that may contribute to these disparities. Additionally, future studies could aim to identify effective interventions or strategies for improving sleep health in both boys and girls, with a particular focus on addressing any gender-specific challenges or barriers that may be present. Ultimately, a better understanding of the gender-specific factors affecting sleep health in childhood could have important implications for promoting healthy sleep habits and overall wellbeing in children of all genders.

We observed better sleep quality overall in early childhood than in middle childhood. The explanation for these differences could be the hormonal component of growth maturation. Sex hormones, estrogen, and progesterone, primarily in females, as well as testosterone, mostly in males, have been shown to have a certain influence on sleep quality [11]. Their levels change at different stages of life, with significant increases happening during puberty. Typically, males start puberty between the ages of 9 to 14 years, while females start between 8 to 12 years old. It takes around three to four years for both sexes to reach full maturity after puberty begins [33]. As it can be noted, the ages of hormonal influence match in some way with the second maturational stage analyzed in this study. This coincidence could explain the maturational stage differences in children’s sleep quality. Additionally, the earlier hormonal influence in females could be be cause of the sex differences observed.

This study has notable strengths, such as (1) the size of its sample, which is noteworthy and may provide some assurance regarding the validity of the results obtained, as well as (2) the recruitment of individuals from a big amount of different primary schools, which allows the knowledge of the sleep and physical habits from a big region’s childhood. However, certain limitations can be taken into consideration. First, we did not consider other potential confounders in the analyses, such as training schedules, nutrition, or screen time. Second, someone could argue that the study lacks objective data on activity and sleep. However, research has shown that subjective information regarding sleep quality is consistent with results obtained from sleep-electroencephalography recordings [16], and the tools here used to assess sleep quality are validated and supported.

## 5. Conclusions

The analysis of the interaction between physical activity and sleep quality in a big sample of primary school children shows that physical activity has a positive effect. Specifically, during middle childhood, greater physical activity is associated with better sleep quality and less sleep latency. Additionally, this study found that sleep quality is generally better in males than in females and is also better in early than middle childhood. Parents should be aware of all the factors that can affect a child’s ability to have a good night’s sleep. Therefore, educational and health institutions need to establish evidence-based guidelines that are appropriate and up to date in promoting healthy sleep habits in children. These guidelines should convey to parents the suitability of their children’s adherence to physical activity, especially from middle childhood, and should also recommend paying special attention to girls’ sleep. Additionally, educational institutions should promote or improve the implementation of physical activity in the school context in order to benefit children’s sleep quality and, hence, improve their quality of life and well-being.

## Figures and Tables

**Table 1:** Comparative values of physical activity levels and sleep quality according to sex and maturational stage.

	Total	Males		Females		Early Childhood		Middle Childhood	
	(*n* = 954)	(*n* = 429)		(*n* = 525)		(*n* = 437)		(*n* = 517)	
			*IC* 95%		*IC* 95%		*IC* 95%		*IC* 95%
Age (y)	10.5± 1.2	10.4 ± 1.2	10.3–10.5	10.6 ± 1.2	10.4–10.6	9.4 ± 0.7	9.4–9.5	11.4 ± 0.6 #	11.3–11.4
Physical activity (1–5)	3.0 ± 0.7	3.1 ± 0.7	3.1–3.2	3.0 ± 0.7 *	2.9–3.0	3.1 ± 0.7	3.0–3.1	3.0 ± 0.7	3.0–3.1
Sleep Quality									
Quality (0–3)	0.7 ± 0.7	0.6 ± 0.7	0.6–0.7	0.8 ± 0.7 *	0.7–0.8	0.6 ± 0.7	0.5–0.7	0.8 ± 0.8 #	0.8–0.9
Latency (0–3)	0.5 ± 0.7	0.4 ± 0.6	0.4–0.5	0.6 ± 0.7 *	0.5–0.6	0.4 ± 0.6	0.4–0.5	0.6 ± 0.7 #	0.5–0.6
Duration (0–3)	0.0 ± 0.2	0.1 ± 0.3	0.0–0.1	0.0 ± 0.2	0.0–0.0	0.0 ± 0.2	0.0–0.1	0.0 ± 0.3	0.0–0.1
Efficiency (0–3)	0.1 ± 0.4	0.1 ± 0.4	0.1–0.2	0.1 ± 0.4	0.1–0.2	0.1 ± 0.4	0.1–0.2	0.1 ± 0.4	0.1–0.2
Disturbance (0–3)	1.3 ± 0.6	1.2 ± 0.6	1.1–1.2	1.4 ± 0.6 *	1.3–1.4	1.3 ± 0.6	1.2–1.4	1.3 ± 0.6	1.2–1.4
Daytime Dysfunction (0–3)	0.5 ± 0.6	0.5 ± 0.7	0.5–0.6	0.5 ± 0.6	0.4–0.5	0.5 ± 0.7	0.4–0.5	0.5 ± 0.6	0.5–0.6
Global PSQI (0–21)	3.0 ± 2.0	3.0 ± 2.0	2.8–3.2	3.4 ± 1.9 *	3.2–3.5	3.0 ± 1.9	2.8–3.1	3.4 ± 2.0 #	3.2–3.6

Mean ± DS. *IC* 95% = Confidence interval to 95%. * = *p* < 0.05 females vs. males. # = *p* < 0.05 middle childhood vs. early childhood.

**Table 2:** Interaction between physical activity, sex, and maturational stage and their mediation on sleep quality.

	PA Levels Early Childhood 8–10 Years			PA Levels Middle Childhood 10–12 Years			PA Levels Early Childhood 8–10 Years			PA Levels Middle Childhood 10–12 Years			*p* Value						
	1st Tertil	2nd Tertil	3rd Tertil	1st Tertil	2nd Tertil	3rd Tertil	1st Tertil	2nd Tertil	3rd Tertil	1st Tertil	2nd Tertil	3rd Tertil	Sex	MS	PA	Sex* MS	Sex * PA	MS * PA	Sex* MS * PA
N	22	120	66	22	139	60	38	134	57	38	200	58							
PA	1.8 ± 0.4	2.9 ± 0.3	4.0 ± 0.3	1.9 ± 0.3	3.0 ± 0.4	4.0 ± 0.3	1.9 ± 0.3	2.9 ± 0.4	4.0 ± 0.3	1.9 ± 0.2	2.9 ± 0.3	3.8 ± 0.3							
Sleep Quality																			
Quality	0.5 ± 0.6	0.6 ± 0.6	0.4 ± 0.7	1.0 ± 0.7	0.7 ± 0.8	0.8 ± 0.7	0.7 ± 0.7	0.7 ± 0.7	0.6 ± 0.8	0.9 ± 0.7	0.9 ± 0.7	0.8 ± 0.8	0.072	0.000	0.469	0.317	0.807	0.235	0.629
Latency	0.3 ± 0.5	0.4 ± 0.6	0.3 ± 0.5	0.7 ± 0.7	0.5 ± 0.7	0.5 ± 0.6	0.5 ± 0.5	0.6 ± 0.7	0.4 ± 0.6	0.9 ± 0.8	0.6 ± 0.7	0.6 ± 0.7	0.004	0.000	0.131	0.742	0.827	0.017	0.982
Duration	0.0 ± 0.0	0.0 ± 0.2	0.1 ± 0.2	0.1 ± 0.3	0.1 ± 0.4	0.0 ± 0.3	0.0 ± 0.0	0.0 ± 0.3	0.0 ± 0.1	0.0 ± 0.2	0.0 ± 0.2	0.0 ± 0.3	0.221	0.242	0.719	0.492	0.964	0.515	0.347
Efficiency	0.0 ± 0.0	0.1 ± 0.4	0.2 ± 0.4	0.1 ± 0.5	0.2 ± 0.5	0.1 ± 0.2	0.2 ± 0.4	0.1 ± 0.4	0.1 ± 0.3	0.2 ± 0.4	0.1 ± 0.4	0.1 ± 0.3	0.384	0.549	0.272	0.962	0.151	0.201	0.231
Disturb.	0.9 ± 0.8	1.2 ± 0.6	1.3 ± 0.6 *	1.3 ± 0.6	1.2 ± 0.6	1.1 ± 0.5	1.5 ± 0.7	1.3 ± 0.6	1.4 ± 0.6	1.5 ± 0.8	1.3 ± 0.6	1.6 ± 0.5 **	0.000	0.262	0.353	0.846	0.031	0.151	0.029
DDysf.	0.3 ± 0.6	0.5 ± 0.7	0.4 ± 0.8	0.5 ± 0.7	0.6 ± 0.7	0.6 ± 0.7	0.5 ± 0.6	0.4 ± 0.5	0.5 ± 0.7	0.4 ± 0.5	0.5 ± 0.6	0.5 ± 0.6	0.643	0.186	0.678	0.116	0.360	0.836	0.673
Global PSQI	2.0 ± 2.0	2.9 ± 1.9	2.6 ± 1.9	3.8 ± 2.3	3.2 ± 2.0	3.0 ± 1.8	3.3 ± 1.8	3.2 ± 1.8	3.0 ± 1.8	4.0 ± 2.4	3.4 ± 1.8	3.6 ± 2.0	0.001	0.000	0.629	0.251	0.307	0.044	0.315

Mean ± DS. Level of significance *p* < 0.05; * *p* < 0.05 vs. 1er tercil of physical activity level. ** *p* < 0.05 vs. 2nd tercil of physical activity level. PA = Phisical Activity. PA levels: 1st tertil. low PA; 2nd tertil. medium PA; 3rd tertil. high PA. Disturb. = disturbance; DDysf = daytime dysfunction. Global PSQI = total Pittsburgh Sleep Quality Index scores. MS = maturational stage. Sex * PA = sex vs. physical activity; MS * PA = maturational stage vs. physical activity; Sex * MS * PA = sex vs. maturational stage vs. physical activity

## Data Availability

The data presented in this study are openly available in [https://osf.io/dnjbp/] at [DOI 10.17605/OSF.IO/DNJBP].
